# Dissecting the role of *CSF2RB* expression in human regulatory T cells

**DOI:** 10.3389/fimmu.2022.1005965

**Published:** 2022-12-02

**Authors:** Beatriz F. Côrte-Real, Rebeca Arroyo Hornero, Aleksandra Dyczko, Ibrahim Hamad, Markus Kleinewietfeld

**Affiliations:** ^1^ Vlaams Instituut voor Biotechnologie (VIB) Laboratory of Translational Immunomodulation, Vlaams Instituut voor Biotechnologie (VIB) Center for Inflammation Research (IRC), Hasselt University, Diepenbeek, Belgium; ^2^ Department of Immunology, Biomedical Research Institute, Hasselt University, Diepenbeek, Belgium; ^3^ University Mulpitle Sclerosis Center (UMSC), Hasselt University (UHasselt)/Campus, Diepenbeek, Belgium

**Keywords:** CSF2RB, CD4 + T cells, FOXP3 + Tregs, autoimmunity, MS

## Abstract

Colony stimulating factor 2 receptor subunit beta (*CSF2RB*; CD131) is the common subunit of the type I cytokine receptors for granulocyte-macrophage colony-stimulating factor (GM-CSF), interleukin (IL)-3 and IL-5. Interestingly, FOXP3^+^ regulatory T cells (Tregs), which play a pivotal role in prevention of autoimmunity have been demonstrated to highly overexpress *CSF2RB* and genome-wide association studies (GWAS) identified *CSF2RB* as being linked to autoimmune diseases like multiple sclerosis (MS). However, the exact biological role of CD131 in human Tregs has not been defined yet. Here we investigated CD131 importance on Treg phenotype and function in a broad range of *in vitro* studies. Although we could not recognize a specific function of *CSF2RB*; CD131 in human Tregs, our data show that CD131 expression is vastly restricted to Tregs even under stimulatory conditions, indicating that CD131 could aid as a potential marker to identify Treg subpopulations from pools of activated CD4^+^ T cells. Importantly, our analysis further demonstrate the overexpression of *CSF2RB* in Tregs of patients with autoimmune diseases like MS and systemic lupus erythematosus (SLE) in comparison to healthy controls, thereby indicating that *CSF2RB* expression in Tregs could serve as a potential novel biomarker for disease.

## Introduction

Regulatory FOXP3^+^ T cells (Tregs) are indispensable for immune homeostasis, playing a crucial role in suppression of exacerbated inflammatory immune responses and maintaining immune tolerance (1, 2), Accumulating evidence indicates reduced Treg numbers and compromised function in autoimmune diseases such as multiple sclerosis (MS), type 1 diabetes (T1D) or systemic lupus erythematosus (SLE) (3–6). Importantly, Treg manipulation can effectively improve disease outcomes in experimental animal models of autoimmunity, and clinical trials are underway examining Treg adoptive cell therapy for the treatment of e.g. T1D, autoimmune hepatitis, Crohn’s disease (CD) and cutaneous lupus and pemphigus (NCT02691247, NCT02772679, NCT02704338, NCT03185000, NCT02428309 and NCT03239470).

Treg function depends on the stable expression of the transcription factor FOXP3 (7), which is one of the most suitable markers to identify Tregs from conventional CD4^+^ T cells (Tconvs). Numerous studies have helped to define a specific Treg gene expression signature which further assists in the detection of particular markers that solely identify Tregs and to pinpoint new functional molecules involved in Treg stability and function (8–11). In this regard, colony stimulating factor 2 receptor subunit beta (*CSF2RB*), has been repetitively acknowledged to be overexpressed in Tregs compared to CD4^+^ Tconvs, but its role in Treg function still remains to be characterized. Interestingly, certain single nucleotide polymorphisms (SNPs) in *CSF2RB* are associated with inflammatory responses like reaction to mosquito bites and with immune-mediated diseases and autoimmunity including eczema, allergy, inflammatory bowel disease (IBD) and MS, which indicates a crucial role for *CSF2RB* in various diseases (12).

*CSF2RB* encodes for CD131, the common beta-chain of the heterodimeric receptors for the β common chain cytokines granulocyte-macrophage colony-stimulating factor (GM-CSF), interleukin (IL)-3 and IL-5. These receptors are located at the cell surface and consist of an alpha subunit that determines specificity to each cytokine, and a common beta subunit (CD131) as the major component for signal transduction (reviewed by Hercus et al. (13)). Since cytokines and the microenvironment are crucial in regulating Treg stability and function (1, 14–16), the overexpression of *CSF2RB* in Tregs is of particular interest and it is possible that CD131 may plays a role as sensor of the cytokine environment, impacting Treg metabolism and function during immune responses.

CD131 mediates intracellular signaling by activating Janus kinase 2 (JAK2), leading to phosphorylation of specific sites of CD131 and triggering signaling pathways such as STAT5, MAPK and PI3-Kinase/AKT which regulate cell survival, proliferation and function (13). IL-3, IL-5 and GM-CSF are pleiotropic cytokines that stimulate hemopoiesis as well as regulate proliferation and activity of immune cells, but little is known about the relation of these cytokines and Tregs. Expression of the alpha chains of IL-3 (IL-3RA) and GM-CSF (GMRα) receptors have been reported in murine Tregs (17, 18). Zhao et al. recently showed an autocrine effect of IL-3 in Tregs that lead to lessening of their immunosuppressive properties (18). By contrast, GM-CSF may induce Treg proliferation *in vitro* independently of IL-2 and enhance Treg suppressive capacity (17). Despite its description in murine Tregs, there is no significant data on IL-3RA or GMRα expression in human Tregs. It is possible that cytokine receptor expression is inducible by certain environmental cues. For instance, IL-4 treatment increases IL-5RA expression in TCR-activated human Tregs, making IL-5 a potential approach to promote antigen-specific Tregs (19).

In addition, erythropoietin receptor (EPO-R) could be assembled as a homodimer of EPO-R chains or as a heterodimer of EPO-R and CD131 (20, 21). EPO is required for red blood cell development but it also shows immunosuppressive properties, impairing T cell proliferation without affecting Treg function (22). However, the effect of EPO on human T cells is restricted to EPO-R homodimer signaling since treatment with ARA290, a specific agonist peptide for EPO-R/CD131 receptor, did not alter T cell activity (22). Purroy et al. have reported high EPO-R expression on human Tregs and increased STAT5 phosphorylation upon combined IL-2 and EPO stimulation (23), however whether EPO acts *via* EPO-R homodimer or EPO-R/CD131 heterodimer receptors on Tregs is not well studied yet.

Thus, since *CSF2RB* is linked to autoimmunity and inflammation and highly expressed by human Tregs, we here investigated the potential role of *CSF2RB* in human Tregs in detail.

## Materials and methods

### Cell isolation and cell sorting

The study was conducted according to the Declaration of Helsinki principles and was approved by the institutional ethics committee of Hasselt University (Comité voor Medische Ethiek UHasselt, CME2019/042 and CME2016/629). Peripheral blood (collected in Lithium Heparin coated tubes, 455084, Greiner bio-one) or buffy coats (purchased from the Belgium Red Cross) were obtained from healthy donors, after providing written informed consent. Human peripheral blood mononuclear cells (PBMCs) were isolated by Ficoll-Paque PLUS (GE17-1440-02, Sigma-Aldrich) gradient centrifugation. In some cases, CD4^+^ T cells were isolated from whole blood using RosetteSep™ Human CD4^+^ T Cell Enrichment Cocktail (15062, Stemcell Technologies) according to manufacturer’s protocol. CD4^+^ T cells or PBMCs were then incubated with CD25 Microbeads II (130-097-044, Miltenyi Biotec) and separated using LS columns (Miltenyi Biotec). Tregs were isolated from CD25^+^ enriched cells, while Tconvs were isolated from CD25-depleted cells. Cells were labelled with Propidium Iodide (PI) (556463, BD Biosciences) prior being sorted as PI^-^CD4^+^CD25^+^CD127^-^ Tregs or as PI^-^CD4^+^CD25^-^CD127^+^ Tconvs by FACS using a BD FACSAria II cell sorter and anti-CD4 APC-Cy7 (clone RPA-T4, 557851, BD Biosciences), CD25 PE-Cy7 (clone M-A251, 557741, BD Biosciences) and CD127 PerCP-Cy5.5 (clone A019D5, 351322, Biolegend) antibodies.

### Cell culture

Human Tconvs and Tregs were cultured in X-VIVO15 media (BE02-060F, Lonza) supplemented with 5% heat inactivated Fetal Bovine Serum (FBS) (S1400, BioWest). Cells were stimulated with 1-10µg/ml plate bound anti-CD3 mAb (555329, BD Biosciences) and 1µg/ml soluble anti-CD28 mAb (555725, BD Biosciences). Alternatively, Treg inspector beads (anti-CD2/CD3/CD28 mAb-coated beads) (130-092-909, Miltenyi Biotec) at 1 bead:1 cell ratio were used for cell stimulation. Media was supplemented with 25-300U/ml IL-2 (11147528001, Sigma-Aldrich). In some cases, Proleukin^®^ (Novartis) was used instead of IL-2 at 5 times higher concentration. When stated, medium was supplemented with additional cytokines IL-4 (11340045, Immunotools), TGFβ (14-8348-62, eBioscience), IL-6 (206-IL, R&D Systmens), IL-1β (201-LB-005, R&D Systems), IL-10 (217-IL-005, R&D Systems), IL-3 (200-03, PeproTech), IL-5 (200-05, PeproTech) or GM-CSF (300-03, PeproTech).

### Quantitative polymerase chain reaction with reverse transcription

Cells were lysed in RLT buffer (Qiagen) and stored at -80°C until RNA was extracted. RNA was isolated using the RNeasy plus Micro Kit (Qiagen) according to the manufacturer’s instructions and further converted to cDNA using qScriptTM cDNA SuperMix kit (QuantaBio) according to manufacturer’s instructions. Real Time PCR was performed on a Step ONE Plus RT-PCR machine (Applied Biosciences) using the TaqMan Fast Universal PCR Master Mix (ThermoFisher Scientific). The following primers were used for PCR: *CSF2RB*- Hs01036514_m1, *CSF2RA*-Hs00531296_g1, *IL3RA*-Hs00608141_m1, *IL5RA*-Hs00602482_m1, *IL10*-Hs00961622_m1, *EPOR*-Hs00959427_m1 (ThermoFisher Scientific) and *B2M*-4326319E-1402015 (Applied Biosystems). Fold-changes in expression were calculated using the ΔΔCT method using human *B2M* as an endogenous control for mRNA expression as described before (24).

### Flow cytometry (FACS)

For flow cytometry experiments, cells were stained with propidium iodide (PI) (556463, BD Biosciences) or LIVE/DEAD kit (L34976, Invitrogen) according to manufacturer’s instructions to exclude dead cells. For surface staining, cells were labelled with respective antibodies for 20 minutes in MACS buffer (PBS with 0.5% BSA, 2mM EDTA) at 4°C. For intracellular staining, cells were first fixed and made permeable using eBioscience™ Foxp3/Transcription Factor Staining Buffer Set (00-5523-00, Invitrogen) according to manufacturer’s instructions and stained with respective antibodies diluted in Perm buffer for 30 minutes at 4°C, washed and assayed in MACS buffer. For intracellular cytokine staining, cells were stimulated with 50ng/ml phorbol12-myristate13-acetate (PMA) and 250ng/ml Ionomycin (Sigma) in the presence of GolgiPlug (BD) for 5 hours. Data were acquired on a BD LSR Fortessa II and analyzed with FlowJo software (TreeStar). Antibodies used were CD4 APC-Cy7 (557851, BD Biosciences), CD4 PerCP-Cy5.5 (300530, Biolegend), CD3 Pacific Blue (317314, Biolegend), CD25 PE-Cy7 (557741, BD Biosciences), CD127 PerCP-Cy5.5 (351322, Biolegend), CD131 PE (306104, Biolegend), IL-3RA (563599, BD Biosciences), FOXP3 PE (320108, Biolegend), FOXP3 AF700 (56-4776-41, eBioscience™), IL-2 APC (17-7029-82, eBioscience™), IFNɣ FITC (11-7319-82, eBioscience™), TNFα eF450 (48-7349-42, eBioscience™), IL-17A PerCP-Cy5.5 (45-7179-42, eBioscience™). When indicated, cells were stained with the cell trace dyes CFSE (C34554, ThermoFisher Scientific) or CTV (C34557, ThermoFisher Scientific), at a final concentration of 1µM or 2.5µM respectively.

### Phosphorylation assays

Tregs were stimulated *in vitro* with 1µg/ml plate bound anti-CD3 mAb (555329, BD Biosciences), 1µg/ml soluble anti-CD28 mAb (555725, BD Biosciences) and 25U/ml IL-2 (11147528001, Sigma-Aldrich) for 4 days. Cells were then rested for 2 hours previous a 2 hours stimulation with 20 or 100 ng/ml GM-CSF (300–03), IL-5 (200-05) or IL-3 (200-03) (PeproTech). As a positive control, Tregs were stimulated with 100U/ml IL-2 (11147528001, Sigma-Aldrich). Cells were fixed with BD Cytofix™ Fixation Buffer (554655, BD Biosciences) for 10 minutes at 37°C and permeabilized with Perm Buffer III (558050, BD Biosciences) for 30 minutes on ice. Cells were stained with anti-pSTAT5 antibody (560311, BD Biosciences) for 30 minutes at 4°C and were acquired on a BD LSR Fortessa II.

### gRNA activity testing in HEK cells

gRNAs targeting *CSF2RB* were designed and tested in HEK293T cells for their *in vitro* effectiveness of creating indels as described before (25). Briefly, HEK293T cells were transfected using jetOptimus buffer (Polyplus, #117-07) with 300 ng Cas9 plasmid (pU6-(BbsI)Cbh-Cas9-T2A-mCherry; Addgene plasmid #64324) and 150 ng OOF plasmid (pBS SK mCherryROSAegfp; Addgene plasmid #54322) according to manufacturer’s protocol and incubated at 37°C for 48 hours before flow cytometry read-out. gRNAs were considered working when at least 33% of the transfected cells were GFP^+^.

### Epigenetic data

Histone epigenetic marks and chromHMM data from the Roadmap Epigenomics Project (26) were downloaded from WashU EpiGenome Browser (v52.1.0) (27). Data were extracted from human CD4^+^CD25^-^ Th and CD4^+^CD25^+^CD127^-^ Treg primary cells isolated from blood of healthy donors (donors 62 and 332) as described before (28).

### Reanalysis of published transcriptomic datasets

EGAS00001004470, GSE90600, GSE76598 were downloaded from the European Genome-phenome Archive (EGA) or gene expression omnibus (GEO) database. GSE76598 was analyzed with GEO2R. For reanalysis of RNA sequencing in EGAS00001004470, GSE90600, quality of raw sequence reads was checked using FastQC version 0.11.8, and nucleotide calls with a quality score of 28 or higher were considered high quality. Adapters were removed using cutadapt v.2.4. Reads were aligned to the hg19 genome reference using STAR (2.5.0e) and a maximum of five mismatches were allowed. Gene counts were retrieved using htseq-count using the “union” option as described before (28).

### Genome-wide association study data

Publicly available GWAS catalogue data were obtained from the NHGRI-EBI website (https://www.ebi.ac.uk/gwas/) (12).

### CRISPR/Cas9 genome editing in human Tregs

Genome editing of human Tregs was performed as before (29). Briefly, Treg cells were *in vitro* cultured for 6-7 days prior nucleofection. Cells were stimulated in X-VIVO15 media (BE02-060F, Lonza) supplemented with 5% heat inactivated Fetal Bovine Serum (FBS) (S1400, BioWest) with 10µg/ml plate bound anti-CD3 mAb (555329, BD Biosciences), 1µg/ml soluble anti-CD28 mAb (555725, BD Biosciences) and 300U/ml IL-2 (11147528001, Sigma-Aldrich) or 1500IU/ml Proleukin^®^ (Novartis). Cells were rested 24 hours prior nucleofection in media containing 100U/ml IL-2 (or 500IU/ml Proleukin^®^) in the absence of TCR stimulation. 20pmol Cas9 nuclease (9212-0.25MG, Aldevron) was incubated with 100pmol gRNA (Synthego, **Supplementary Table 1**) for a minimum of 10 minutes at 37°C for RNP complexing. 1 million Tregs were transfected in 20 µl P3 Primary Cell 4D-Nucleofector X Kit S (V4XP-3032, Lonza) with RNPs by using Nucleofection cuvette strips (4D-Nucleofector X Kit S, Lonza) and the 4D-Nucleofector Core Unit (AAF-1002B, Lonza) and X Unit (AAF-1002X, Lonza) with program EO115. Mock control cells were nucleofected with water or Cas9 only in the absence of gRNA. After transfection, Tregs were cultured in X-VIVO15 media supplemented with 5% FBS and 100U/ml IL2 (or 500IU/mL Proleukin^®^) and incubated at 37°C.

### Suppression assays

Treg suppressive capacity was assessed by their ability to suppress autologous T cell proliferation *in vitro* and was done as described before (28, 29). In brief, freshly isolated PBMCs or CD4^+^ Tconvs responder cells (Tresp) were frozen on FBS containing 10% DMSO for later use. On the day of the assay, PBMCs or CD4^+^ Tconvs were thawed and stained with CellTrace CFSE Cell Proliferation Kit (C34554, Thermo Fisher) at 1µM and cultured at 50.000 cells/well with variable number of Tregs in 96-well U-bottom plates in X-VIVO media containing 5% FBS. Cells were stimulated for 4 days using Treg Suppression Inspector beads (130-092-909, Miltenyi Biotec) at 1 bead: 1 cell, or by co-culturing with allogenic monocyte-derived dendritic cells (DCs) (20.000 cells/well) in the presence of 1µg/ml anti-CD3 mAb (555329, BD Biosciences) before FACS analysis. Cell proliferation was calculated using FlowJo software (Treestar) as percentage of Tresp that diluted CFSE. Data is represented after normalizing to control conditions when Tresp were stimulated in the absence of Tregs. For monocyte-derived DCs, CD14^+^ monocytes were magnetic bead-isolated from PBMCs (17858, StemCell Technologies) and cultured with 50U/ml IL-4 (11340045, Immunotools) and 50ng/ml GM-CSF (300-03, PeproTech) in X-VIVO media supplemented with 10% FBS. After 5 days of incubation, DCs were harvested and stored in liquid nitrogen for later use.

### Quantification and statistical analysis

Graphs were produced and statistical analyses were performed with GraphPad Prism Version 8. All data were presented as mean with standard deviation (SD). Each dot displayed in the figures denotes an independent biological donor. Number of donors (n) and statistical tests that were used can be found in figure legends. Paired tests were selected when comparing a cell subset over time or under certain treatments, like comparison of gene expression at different time points or Mock and KO-Tregs from same biological donor. Unpaired tests were selected when comparing between different cell subsets such as Tconvs and Tregs. Significance was defined as *p ≤ 0.05, **p ≤ 0.01, ***p ≤ 0.001 and ****p ≤ 0. 0001.

## Results

### CD131 is overexpressed in resting and activated human Tregs compared to CD4^+^CD25^-^ Tconvs

**Figure 1:**
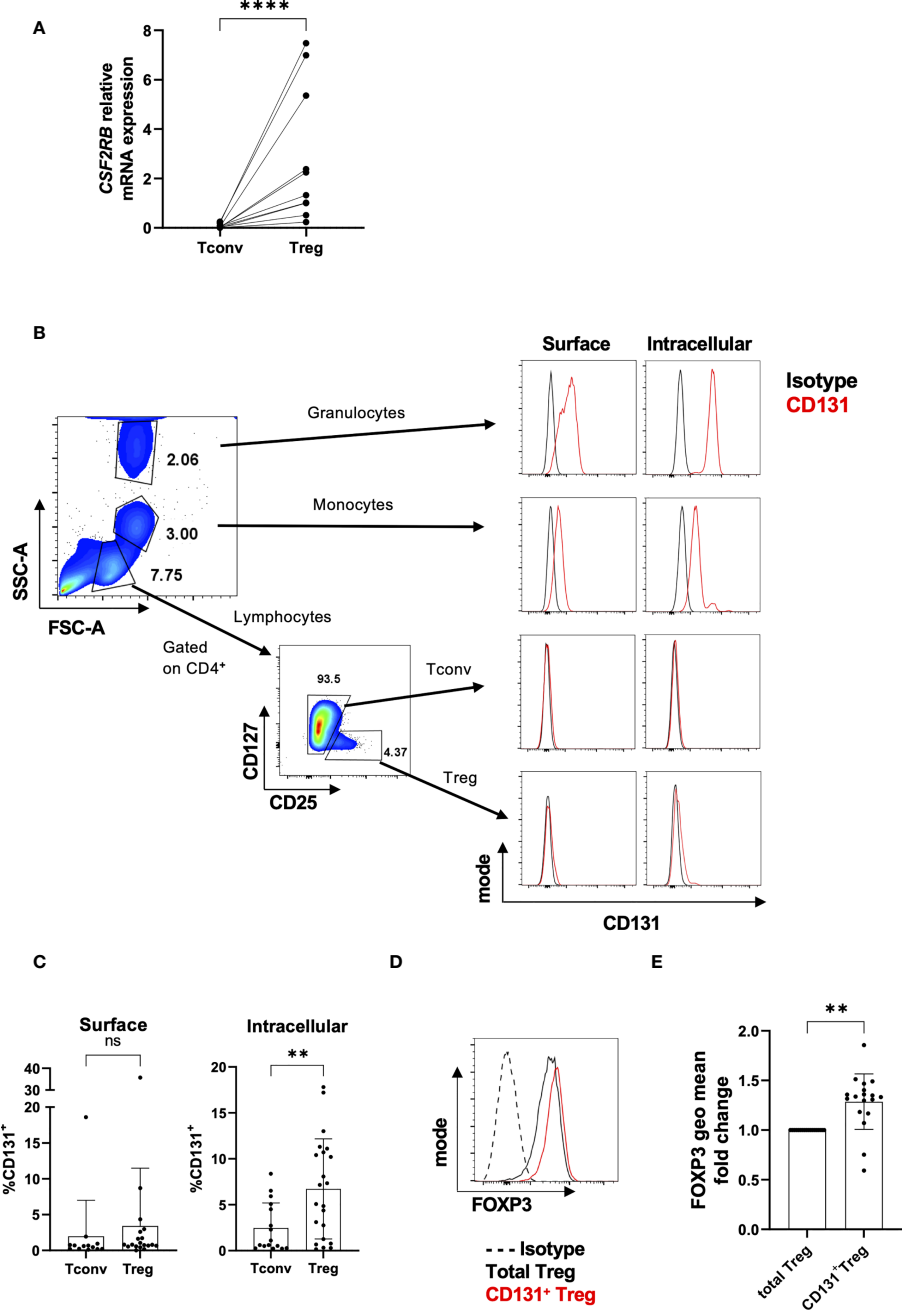
Differential CD131 expression in human CD4^+^CD25^-^ Tconvs and Tregs. **(A)** Relative *CSF2RB* mRNA levels in flow-sorted CD4^+^CD25^-^CD127^+^ Tconvs and CD4^+^CD25^+^CD127^-^ Tregs from freshly isolated human PBMCs (n= 10). The relative quantities of *CSF2RB* mRNA were normalized to the expression of *B2M* mRNA. **(B)** Granulocytes, monocytes and lymphocytes were gated based on forward and side scatter characteristic of cells from cells obtained from healthy human donors. Tconvs and Tregs were further gated based on CD4, CD25 and CD127 expression. Histograms show CD131 expression (red line) in comparison with matched isotype control (black line) for each cell type. One representative donor out of two. **(C)** CD131 surface and intracellular protein expression was studied in CD4^+^CD25^-^CD127^+^ Tconvs and CD4^+^CD25^+^CD127^-^ Tregs in 13-22 independent donors. **(D, E)** FOXP3 expression was analyzed within the total Treg population or within CD131^+^ Tregs. **(D)** Representative FACS plot for one donor. **(E)** FOXP3 fluorescence intensity in CD131^+^ Tregs normalized to the total Treg population (n=18 independent donors). Graphs display mean ± SD. Normal distribution was assessed by Shapiro-Wilk normality test with a significance level of 0.05. Significance was calculated by **(A, C)** Mann-Whitney test or **(E)** Wilcoxon matched-pairs test. ns for P > 0.05, ** for P ≤ 0.01, **** for P ≤ 0.0001.

To investigate the expression of *CSF2RB* in human Tconvs and Tregs we first analyzed its expression in freshly highly pure FACS-sorted Tregs compared to CD4^+^CD25^-^ Tconvs (**Figure S1**) by qRT-PCR. We indeed confirmed that *CSF2RB* is highly overexpressed in Tregs compared to Tconvs (**Figure 1A**). Further, the transcriptional pattern correlated well with the epigenetic status of *CSF2RB* in Tconvs and Tregs, which was indicated to be quiescent/low or strongly transcribed, respectively, when studied using the WashU epigenome browser on two datasets (**Figure S2A**).

We next investigated protein expression of CD131 (encoded by the *CSF2RB* gene) in freshly isolated PBMCs. Previous data in human donors showed low *CSF2RB* transcription along with low CD131 protein expression in e.g. intestinal T cells or T cells from PBMCs compared to other immune cell subsets such as eosinophils, monocytes, macrophages and dendritic cells (https://www.proteinatlas.org/ENSG00000100368-CSF2RB/immune+cell) (30, 31) (**Figure S2B**). In line with these data, we found that CD131 protein expression levels were much higher in granulocytes or monocytes compared to Tregs, whereas CD131 expression in CD4^+^CD25^-^ Tconvs was virtually absent (**Figure 1B**). Accordingly, and in line with the gene expression data, freshly isolated Tregs exhibit a significantly higher intracellular CD131 protein expression compared to CD4^+^CD25^-^ Tconvs, although only a tendency was observed in cell surface expression (**Figure 1C**).

Interestingly, CD131^+^ Tregs displayed higher FOXP3 expression than the total Treg population (**Figures 1D, E** **)**. Higher FOXP3 expression has been previously identified in CD45RA^-^CD25^hi^ Treg subset (32) and transcriptomic studies have shown higher *CSF2RB* mRNA expression in CD45RA^-^ memory-like Tregs (which usually makes up the majority of Tregs from PBMCs) compared to CD45RA^+^ naïve-like Tregs (10, 11) (**Figures S3A, B**). These findings indicate that the majority of CD131^+^ Tregs may cluster in between the CD45RA^-^ memory-like Treg compartment with high FOXP3 expression.

**Figure 2:**
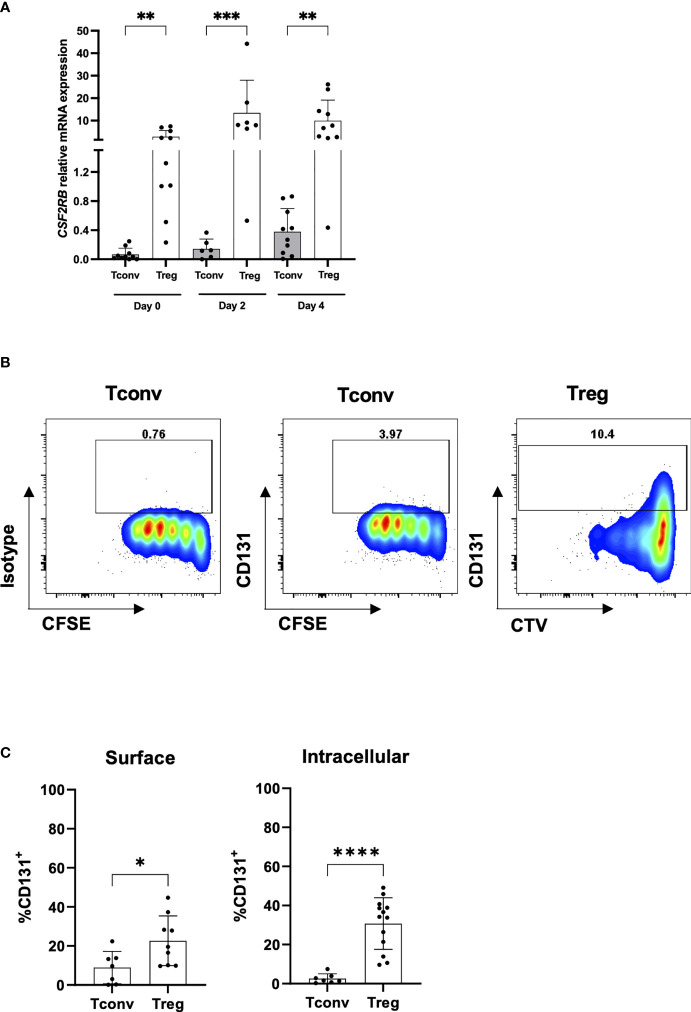
CD131 expression on TCR-stimulated CD4^+^CD25^-^ Tconvs and Tregs. **(A)** Relative *CSF2RB* mRNA levels in flow-sorted CD4^+^CD25^-^CD127^+^ Tconv and CD4^+^CD25^+^CD127^-^ Treg cells from human PBMCs upon *in vitro* stimulation with anti-CD3/CD28 mAbs and IL-2. Cells were stimulated with 1 or 10µg/ml anti-CD3 mAb, 1µg/ml anti-CD28 mAb and 25 or 300U/ml IL-2 (n= 6-10). The relative quantities of *CSF2RB* mRNA were normalized to the expression of *B2M* mRNA. Graph includes data from **Figure 1A** at day 0 for comparison **(B)** CD131 intracellular protein expression was studied in CD4^+^CD25^-^CD127^+^ Tconvs and CD4^+^CD25^+^CD127^-^ Tregs flow-sorted from human PBMCs, labelled with cell proliferation trackers CFSE or CTV and *in vitro*-stimulated with anti-CD3/CD28/CD2 coated beads for 4 days in the presence of 25U/ml IL-2. One representative donor out of two is shown. **(C)** CD131 surface and intracellular protein expression was studied in CD4^+^CD25^-^CD127^+^ Tconv and CD4^+^CD25^+^CD127^-^ Treg cells stimulated for 4 days using 1µg/ml anti-CD3 mAb, 1 µg/ml anti-CD28 mAb and 25U/ml IL-2 (n= 7-13 independent donors). Graphs display mean ± SD. Normal distribution was calculated by Shapiro-Wilk normality test with a significance level of 0.05. Significance was calculated by **(A)** Kruskal-Wallis test with Dunn’s multiple comparisons test and **(C)** unpaired two-tailed *t* test for normal distributed data. * for P ≤ 0.05 ** for P ≤ 0.01,*** for P ≤ 0.001, **** for P ≤ 0.0001.

Importantly, in contrast to other Treg specific markers like CD25, we found *CSF2RB* being significantly overexpressed in Tregs over CD4^+^CD25^-^ Tconvs also after *in vitro* stimulation at both mRNA (**Figure 2A**) and protein levels (**Figures 2B, C** **)** and was stably observed still after 11 days in culture (data not shown). These data go in line with previous observations regarding *CSF2RB* gene expression in 40 hours TCR-stimulated naive or memory CD4^+^ Tconv and Treg cells (10) (**Figure S3C**).

### Effects of β common chain cytokines on human Tregs

**Figure 3:**
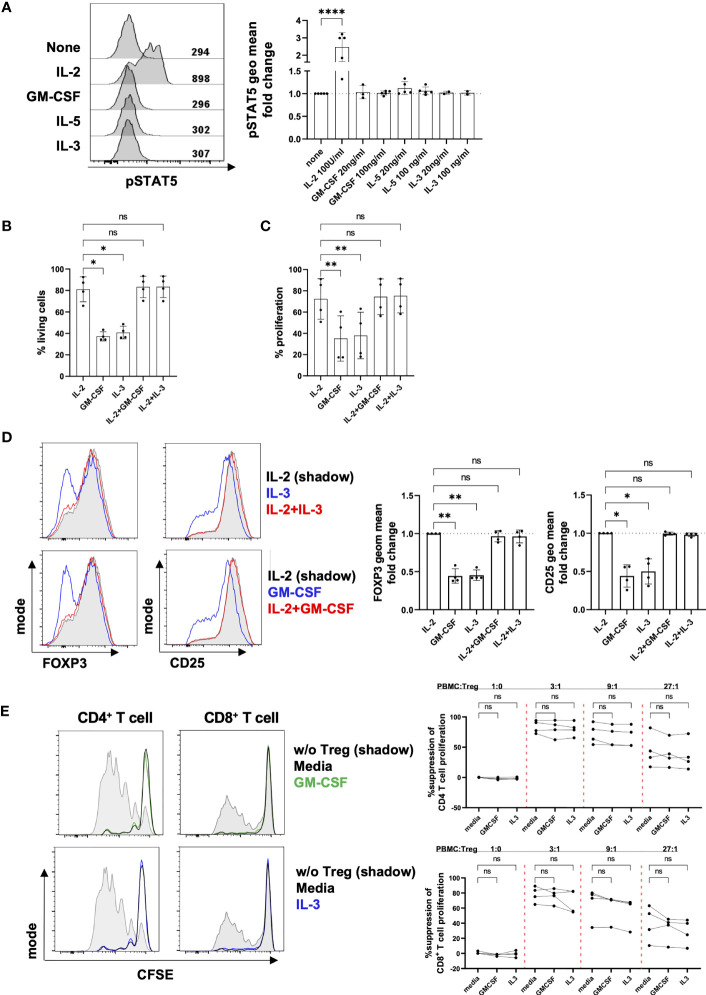
Effect of β common chain cytokines on Tregs. **(A)** After 4 days of *in vitro* stimulation with 1µg/ml anti-CD3 mAb, 1µg/ml anti-CD28 mAb and 25 U/ml IL-2, Tregs were rested for 2 hours in the absence of TCR stimulation and IL-2 prior a 2-hour culture with different cytokines as indicated in the figure. STAT5 phosphorylation was measured by flow cytometry. Fluorescence intensity of pSTAT5 was calculated as geometrical mean (geo mean) and normalized to pSTAT5 in cells cultured in the absence of any cytokine. Left panel: fluorescence intensity of pSTAT5 is shown for 1 representative donor. Right panel: combined data from 2-5 independent donors. **(B-D)** Four day-stimulated Tregs were labelled with CTV proliferation dye and re-stimulated for four further days using anti-CD2/CD3/CD28 mAb-coated beads in the presence of different cytokines as indicated (IL-2: 25U/ml, GM-CSF and IL-3: 100ng/ml). **(B)** Cell viability, **(C)** proliferation **(D)** phenotype and **(E)** *in vitro* suppressive capacity were analyzed by flow cytometry. Treg suppressive activity was assessed by inhibition of proliferation of CFSE-labelled PBMCs when co-cultured with stimulated autologous Tregs in the presence of anti-CD2/CD3/CD28 mAb-coated beads. Cell proliferation was assessed by dilution of CFSE dye in CD4^+^ or CD8^+^ T cells. Tregs were excluded of responder CD4^+^ T cells by gating out CFSE negative cells. Left panel: histograms show data from one representative donor. Grey shadow: T cell proliferation in the absence of Tregs; black line: T cell proliferation when co-cultured with Tregs; green/blue line: T cell proliferation when co-cultured with Tregs in the presence of GM-CSF or IL-3. Right panel: data show mean ± SD of percentage of suppression of cell proliferation for 4 independent donors. Significance was calculated by one-way ANOVA with Holm-Sidak’s multiple comparisons test. ns for P > 0.05, * for P ≤ 0.05, ** for P ≤ 0.01, **** for P ≤ 0.0001.

We next investigated the impact of β common chain cytokines (GM-CSF, IL-5 and IL-3) on Treg function. To ensure CD131 expression at the moment of the functional tests, we assessed the effect of these cytokines on Tregs that were previously stimulated with anti-CD3/CD28 antibodies for 4 days in the presence of IL-2 (**Figure 2C**). CD131 signals through several pathways including JAK2/STAT5 (13, 33). While IL-2, a known activator of STAT5 in T cells, induced STAT5 phosphorylation in Tregs, no effect was observed under GM-CSF, IL-5 or IL-3 treatment (**Figure 3A**). Cell viability and proliferation were not affected by culturing in the presence of a combination of IL-2 and GM-CSF or IL-3, but significantly dropped if GM-CSF or IL-3 were used in the absence of IL-2 (**Figures 3B, C** **)**. Similarly, GM-CSF and IL-3 used in combination with IL-2 did not affect FOXP3 or CD25 expression compared to culture with IL-2 alone (**Figure 3D**). Importantly, the ability of Tregs to suppress CD4^+^ and CD8^+^ T cell proliferation was not altered by the addition of GM-CSF or IL-3 into co-cultures of Tregs with autologous PBMCs (**Figure 3E**). Overall, although CD131 was expressed by Tregs, we did not detect any effect of β common chain cytokines on Treg phenotype or function under the conditions tested.

### Characterization of cytokine-specific alpha subunits on CD4^+^CD25^-^ Tconvs and Tregs

**Figure 4:**
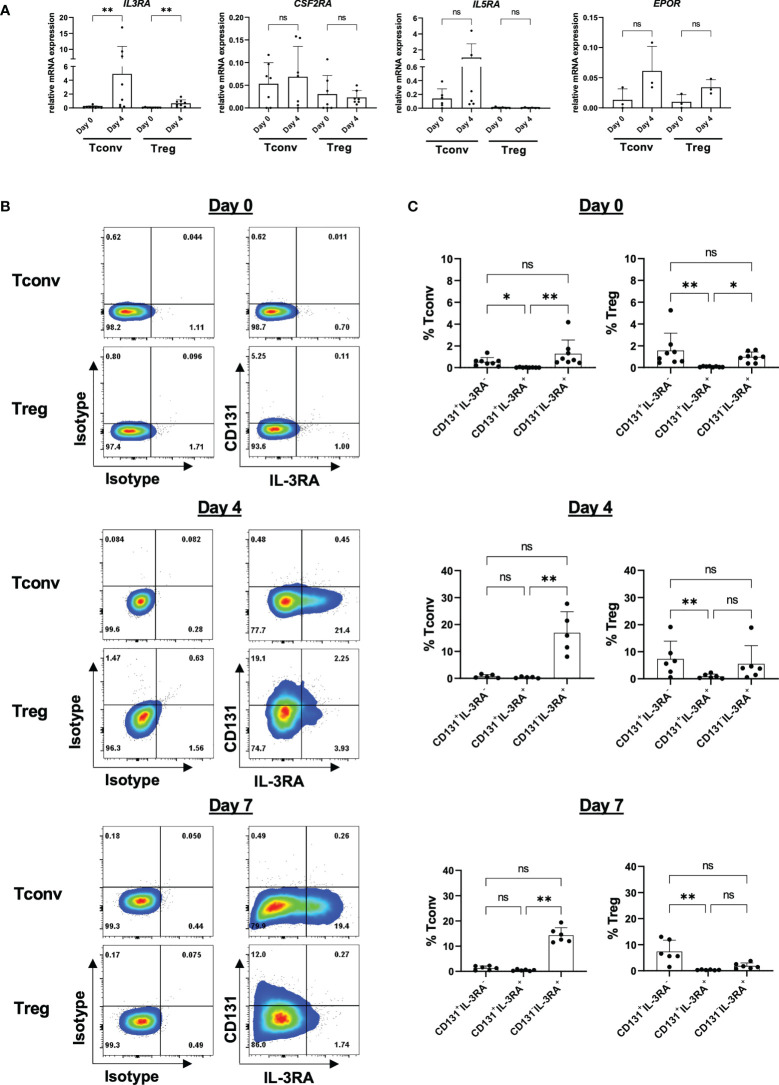
Expression of CD131-associated cytokine receptors on CD4^+^ Tconv and Treg populations. CD4^+^CD25^-^CD127^+^ Tconv and CD4^+^CD25^+^CD127^-^ Treg cells were flow-sorted from human PBMCs and stimulated *in vitro* with 1-10µg/ml anti-CD3 mAb, 1µg/ml anti-CD28 mAb and 25-300U/ml IL-2. **(A)** mRNA expression levels of IL-3 receptor alpha (*IL3RA*), GM-CSF receptor alpha (*CSF2RA*), IL-5 receptor alpha (*IL5RA*) and EPO receptor (*EPOR*) chains were studied in CD4^+^CD25^-^CD127^+^ Tconvs and CD4^+^CD25^+^CD127^-^ Tregs prior (day 0) and after 4 days of *in vitro*-stimulation (day 4). Relative mRNA levels were normalized to the expression of *B2M* mRNA (n=3-8 independent donors). **(B)** IL-3RA and CD131 surface protein expression were studied in CD4^+^CD25^-^CD127^+^ Tconvs and CD4^+^CD25^+^CD127^-^ Tregs prior (day 0) and after 4 or 7 days of *in vitro*-stimulation. One representative donor is shown. **(C)** Cumulative data from 5-8 independent donors. Mean ± SD is shown. Significance was calculated using a Friedman test with Dunn’s multiple comparisons test. ns for P > 0.05, * for P ≤ 0.05, ** for P ≤ 0.01.

CD131 is the shared beta chain of several cytokine receptors and an essential component for cytokine signaling transduction. However, prior CD131 transduction of signaling, the assembly with the alpha chain is essential to build a functional receptor binding to the particular cytokine. Thus, we next analyzed expression of alpha chains of the IL-3 receptor (IL-3RA), GM-CSF receptor (CSF2RA), IL-5 receptor (IL-5RA) and EPO receptor (EPO-R) in Tregs and CD4^+^CD25^-^ Tconvs. However, in contrast to Tconvs, we only found low mRNA expression levels in either freshly isolated Tregs or after 4 days of *in vitro* stimulation (**Figure 4A**) which is again in line with the reanalysis of datasets from previous reports (10, 11) (**Figure S4**).

Amongst the alpha chains tested, IL-3RA showed the highest expression and was further significantly upregulated upon *in vitro* activation in Tregs (**Figure 4A**). We therefore sought to study whether IL-3RA is also expressed at protein level under resting and stimulatory conditions over time (**Figures 4B, C** **)**. Surface IL-3RA protein expression in human TCR-stimulated (but not in resting state) CD4^+^ T cells has been previously reported (34, 35). Moreover, Srivastava et al. described high IL-3RA expression on mouse Tregs isolated from splenocytes (36). Our data corroborated absence of IL-3RA protein expression on freshly isolated human CD4^+^CD25^-^ Tconvs but also on Tregs, and observed preferential IL-3RA expression in TCR-stimulated CD4^+^CD25^-^ Tconvs over Tregs, with no substantial differences between days 4 and 7 of culture (**Figures 4B, C** **)**. Co-expression of alpha and beta subunits is considered to be necessary for the formation of high-affinity receptors (37). However, importantly, the majority of the cells showed CD131/IL-3RA single expression, with only a minor subpopulation co-expressing both CD131 and IL-3RA (**Figures 4B, C** **)**.

**Figure 5:**
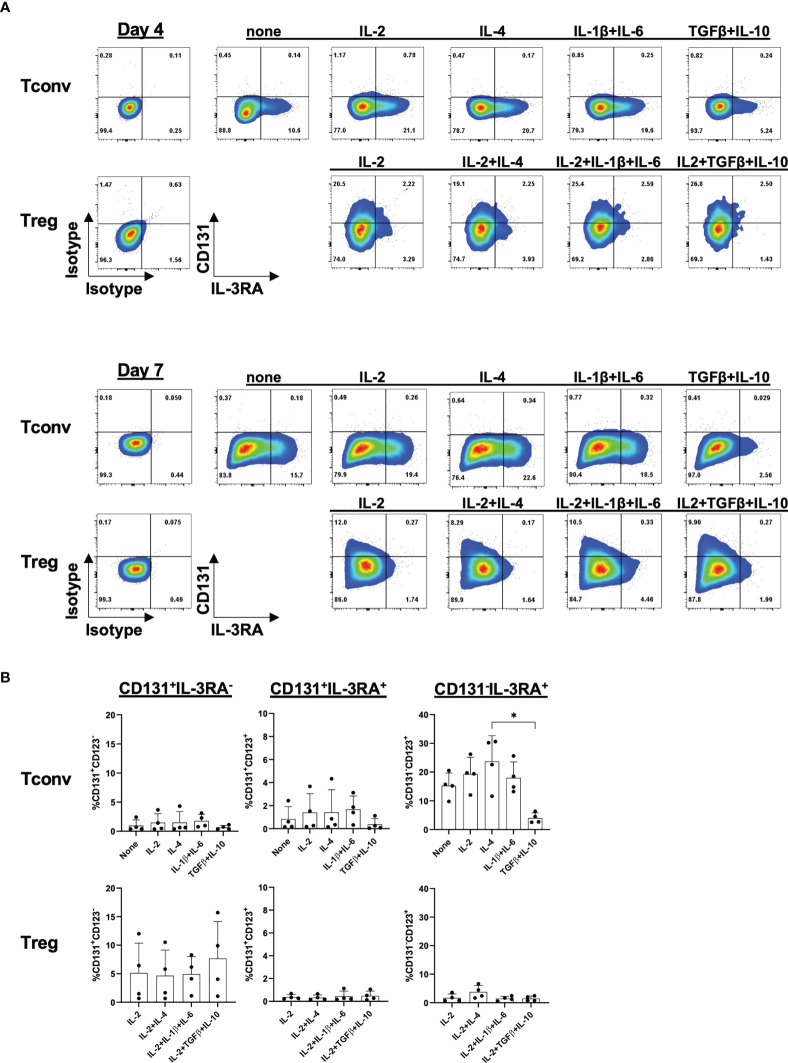
Cytokine-mediated regulation of CD131 and IL-3RA expression. Surface CD131 and IL-3RA expression was studied in CD4^+^CD25^-^ Tconvs or Tregs cultured for 4 or 7 days with 5µg/ml anti-CD3mAb, 1µg/ml CD28 mAb and different cytokines as indicated (IL-4, IL-6, IL-1β and IL-10: 25ng/ml, TGFβ: 10ng/ml). Additional 100U/ml IL-2 was added to all Treg cultures or 25U/ml IL-2 to Tconv where indicated. **(A)** One representative donor out of four is shown. **(B)** Mean ± SD is shown for n=4 independent donors after 7 days of stimulation. Significance was calculated using a Friedman test with Dunn’s multiple comparisons test. Only p-values lower than 0.05 are shown. * for P ≤ 0.05.

Since it is possible that CD131 and IL-3RA expression are regulated by specific cytokine environments, we next investigated the induction of CD131 and IL-3RA under different stimulatory conditions (**Figure 5**). While IL-2 and IL-4 have been previously shown to increase IL-3RA expression, IL-10 reduced the percentage of IL-3RA^+^ cells on *in vitro* activated T cells (34, 35). However, to our knowledge, there is no information about cytokine-mediated regulation of CD131 or IL-3RA expression on human Tregs. To assess whether the cytokine environment could regulate CD131/IL-3RA-expression on human Tregs, CD4^+^CD25^-^ Tconvs and Tregs were activated *in vitro* in the presence of IL-2, IL-4 or a combination of IL-1β and IL-6 or TGFβ and IL-10 and were cultured for 4 or 7 days. However, only the combination of immune suppressive cytokines TGFβ and IL-10 significantly affected the phenotype of CD4^+^CD25^-^ Tconvs compared to IL-4 or IL-2 treatments, by reducing the proportion of CD131^-^IL-3RA^+^ cells, while again no substantial differences were observed in Tregs (**Figures 5A, B** **)**.

Thus, under the conditions tested, only a minority of Tregs seems to express a functional cytokine receptor assembled with the CD131 beta chain.

### Human CSF2RB-KO Tregs display unaltered phenotype and function

**Figure 6:**
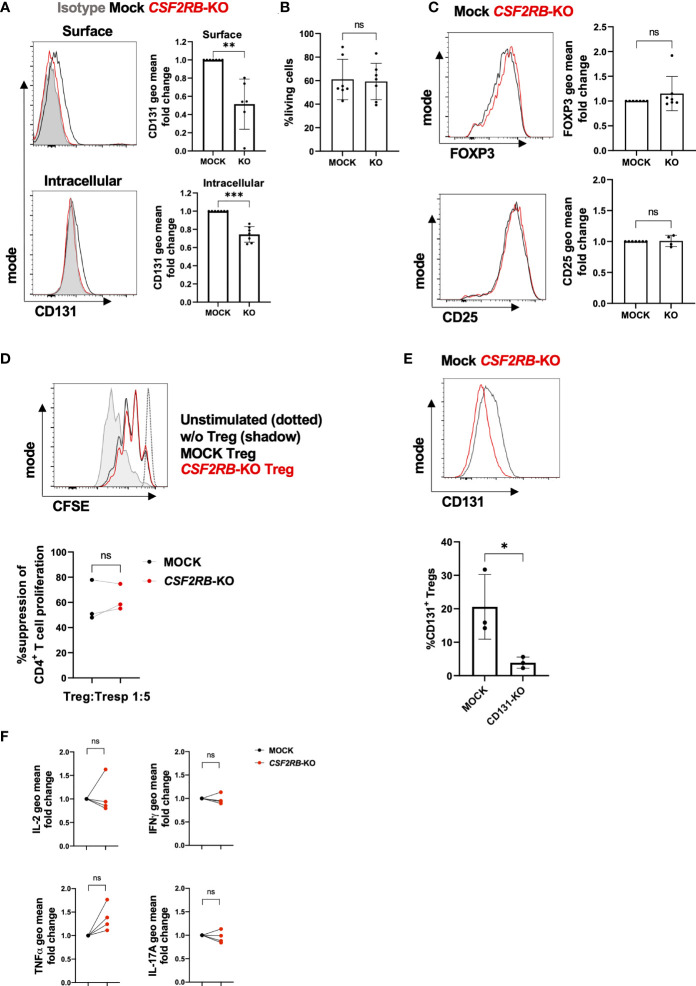
Characterization of *CSF2RB*-KO Tregs**(A–E)** CD4^+^CD25^+^CD127^-^ Tregs were flow-sorted from human PBMCs and cultured *in vitro* for 7 days with 10µg/ml anti-CD3 mAb, 1µg/ml anti-CD28 mAb and 300 U/ml IL-2 prior *CSF2RB* gene knock-out (KO) by Cas9/sgRNA ribonucleoprotein (RNP) transfection or controls (Mock). **(A)** CD131 surface and intracellular expression, **(B)** cell viability and **(C)** FOXP3 and CD25 expression were studied 3-4 days post-electroporation in 4-7 independent donors. Histograms show data from one representative donor. Isotype (if applicable) is shown as a grey shadow, mock as a black line and *CSF2RB*-KO as a red line. **(D)** *In vitro* suppressive capacity of MOCK or *CSF2RB*-KO Tregs was assessed by measuring their ability to inhibit proliferation of CFSE-labelled CD4^+^CD25^-^ Tconv (Tresp) when stimulated with monocyte-derived dendritic cells (moDC) and anti-CD3 mAb. Top panel: CFSE expression for one representative donor. Dotted black line: proliferation of unstimulated Tresp; grey shadow: Tresp proliferation in the absence of Tregs; black line: Tresp proliferation when co-cultured with Mock Tregs; red line: Tresp proliferation when co-cultured with *CSF2RB*-KO Tregs. Bottom panel: percentage of suppression of CD4^+^ Tresp that diluted CFSE dye is represented for 3 independent donors. **(E)** CD131 expression was measured by flow cytometry in Tregs harvested from suppression assay cultures and gated as live, CD3^+^CD4^+^CFSE^-^ cells. **(F)** 4 days after nucleofection, MOCK or *CSF2RB*-KO Tregs were re-stimulated with anti-CD3/CD28 mAbs and IL-2 for 4 days. Tregs were re-stimulated with PMA and ionomycin in the presence of Brefeldin A prior staining for cytokine expression (n=4 independent donors). Mean ± SD is shown for cumulative data. Normal distribution was calculated by Shapiro-Wilk normality test with a significance level of 0.05. Significance was calculated by **(A, B, D, E)** paired two-tailed *t* test for normal distributed data or **(C)** by Wilcoxon matched-pairs test **(F)** one-way ANOVA with Holm-Sidak’s multiple comparisons test. ns for P > 0.05, * for P ≤ 0.05, ** for P ≤0.01, *** for P ≤ 0.001.

To ultimate investigate the role of *CSF2RB* in human Tregs, we disrupted *CSF2RB* expression by genome editing using CRISPR/Cas9 technology (**Figure 6**). CRISPR guide-RNAs (gRNAs) were designed and tested for their *in vitro* targeting efficiency on HEK293T cells and Tregs were stimulated with anti-CD3/anti-CD28 mAbs in the presence of IL-2 prior nucleofection of Cas9/gRNA ribonucleoprotein complexes (RNP) as described before (29). Knock-out (KO) efficiency was analyzed 3 to 4 days after nucleofection by measuring CD131 protein expression by FACS compared to controls (Mock). *CSF2RB*-KO Tregs showed significant decrease of CD131 expression on the cell surface and intracellularly (**Figure 6A**), without changes in viability, FOXP3 or CD25 expression compared to controls (**Figures 6B, C** **)**. The suppressive activity of Tregs was assessed by studying their ability to suppress CD4^+^ Tconv proliferation *in vitro* upon co-culture in the presence of dendritic cells and soluble anti-CD3 mAb. Of note, *CSF2RB*-KO Tregs showed similar suppressive potency as controls (**Figure 6D**) and absence of CD131 expression was further confirmed in *CSF2RB*-KO Tregs when harvested after the suppression assay (**Figure 6E**).

We next investigated potential effects of *CSF2RB*-KO on cytokine expression of Tregs. However, we did not observe significant changes in IL-2, IFN-ɣ, TNF-α or IL-17A cytokine expression, although there was a slight tendency in higher TNF-α expression in *CSF2RB*-KO Tregs (**Figure 6F**). We further detected no differences in *IL10* expression and, as expected, the addition β common chain cytokines (IL-3, GM-CSF or IL-5) did similarly not affect cytokine expression in controls or *CSF2RB*-KO Tregs (data not shown).

Thus, in line with previous experiments, also the KO of *CSF2RB* in human Tregs did not show any substantial effects on phenotype or function of Tregs under the tested conditions.

### *CSF2RB* expression is upregulated in Tregs of patients with autoimmunity

**Figure 7:**
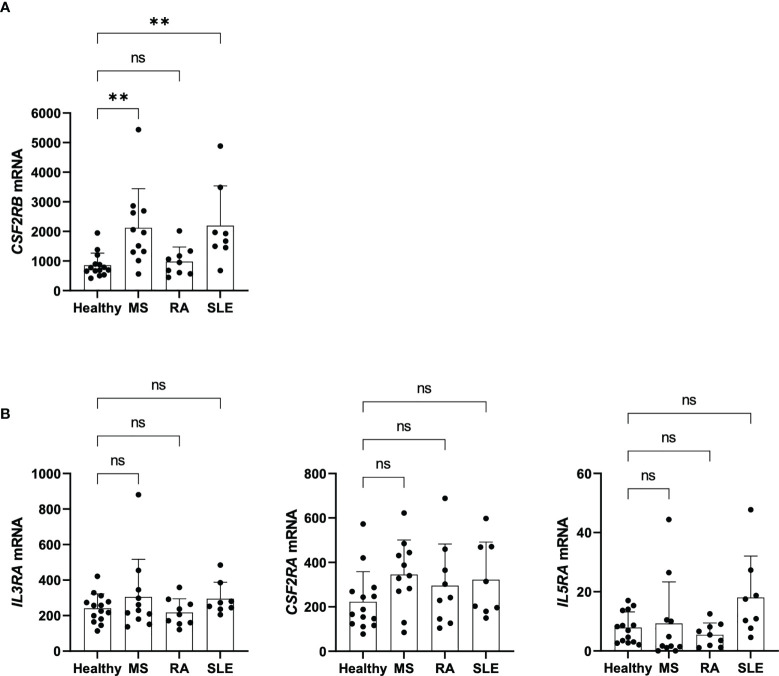
*CSF2RB* expression of Tregs in patients with autoimmunity. **(A)** *CSF2RB* mRNA levels in Tregs isolated from healthy individuals (n= 14), and patients with MS (n= 11), RA (n= 9) and SLE (n= 8). Data obtained and reanalyzed from (3). **(B)** *IL3RA, CSF2RA and IL5RA* mRNA levels in Tregs isolated from healthy individuals (n= 14), and patients with MS (n= 11), RA (n= 9) and SLE (n= 8). Data obtained and reanalyzed from (3). Mean ± SD is shown. Normal distribution was assessed by Shapiro-Wilk normality test with a significance level of 0.05. Significance was calculated by Kruskal-Wallis test with Dunn’s multiple comparisons test. ns for P > 0.05; ** for P ≤ 0.01.

The analysis of genetic data published on genome-wide association studies (GWAS) Catalog from the National Human Genome Research Institute (NHGRI) Catalog shows that *CSF2RB* has susceptibility variants for MS (12) (**Supplementary Table 2**). To determine whether *CSF2RB* expression of Tregs relates to autoimmunity, we re-analyzed published transcriptomic datasets of Tregs isolated from healthy controls and patients with MS, rheumatoid arthritis (RA) and SLE (3). Of note, we found *CSF2RB* to be significantly overexpressed in Tregs of MS and SLE patients compared to healthy controls, although no significant changes were observed in RA patients (**Figure 7A**). As a control we similarly investigated the expression of the alpha chains *IL3RA*, *CSF2RA* and *IL5RA*, but the expression of those genes was not changed (**Figure 7B**). These data indicate that although we could not detect any functional impact, higher *CSF2RB* expression on Tregs could may serve as a novel biomarker for MS and SLE.

## Discussion

Previous studies have identified a human Treg gene expression signature where *CSF2RB* is one of the most significantly overexpressed genes in comparison to CD4^+^ Tconvs. An initial characterization of human *CSF2RB* (https://www.proteinatlas.org/ENSG00000100368-CSF2RB/blood) has shown dominant expression in granulocytes, monocytes and memory Tregs (30). Despite all these observations, the role of *CSF2RB* in Tregs function still remains unknown (8–11). *CSF2RB* has also been linked to autoimmune disorders, with three variants associated with MS: rs5756405, rs5756391 and rs2413436 (12, 38). Although the phenotypic consequences of these single nucleotide polymorphisms (SNPs) are not yet understood, *CSF2RB* mutations have been related to altered GM-CSF signaling and STAT pathways. Loss-of-function mutations in *CSF2RB* have been described in Crohn’s Disease and pulmonary alveolar proteinosis, resulting in defective GM-CSF-mediated STAT5 phosphorylation (31, 39). By contrast, Watanabe-Smith et al. identified a *CSF2RB*-activating mutation in a leukemia patient which resulted in higher protein stability, prolonged accumulation on cell surface and constitutive STAT pathway activation in a ligand independent manner (33). While *CSF2RB* is expressed by Tregs and STAT5 signaling is crucial for Treg biology, there is no evidence yet relating *CSF2RB* alterations in human Tregs with disease.

We have demonstrated *CSF2RB* overexpression in human Tregs compared to CD4^+^ Tconvs in resting and *in vitro*-activated cells and independently confirmed these findings by the reanalysis of previously published datasets. Importantly, upon *in vitro* TCR-stimulation, CD131 protein was almost exclusively found in Tregs, with minimum expression in CD4^+^ Tconvs. These data indicate that CD131 could be a potential marker for identification of Tregs from a pool of activated CD4^+^ Tconvs, for instance during inflammatory conditions since both, FOXP3 and CD25, could be temporary induced in Tconvs as well. Further studies will clarify if CD131 might be valuable marker to specifically identify or isolate Tregs.

Tregs are highly dependent on IL-2 for activation of STAT5 signaling, which has an essential role in Treg function by regulating FOXP3 expression (40–42). Since β common chain cytokines can signal through STAT5 (13, 33), they may have the potential to regulate Treg phenotype and function. However, unlike IL-2, none of the β common chain cytokines GM-CSF, IL-5 or IL-3 were capable of inducing STAT5 phosphorylation in human Tregs in our *in vitro* set up. Moreover, our data demonstrated that IL-2 is required for Treg survival, proliferation and stable FOXP3 expression, while GM-CSF or IL-3 were not able to compensate for the absence of IL-2. Furthermore, Treg *in vitro* suppressive activity was not disturbed in the presence of GM-CSF or IL-3.

CD131 is the shared beta-chain and main signaling component of IL-3, IL-5 and GM-CSF receptors. After cytokine interaction, the ligand specific alpha-chain binds to CD131 leading to the formation of multimeric complexes that induce intracellular phosphorylation cascades (13). Hence, co-expression of CD131 and the alpha-chain is therefore necessary for appropriate cytokine signaling. Here we have found that the alpha-chains IL3RA, IL5RA or CSF2RA are low expressed on resting or *in vitro* activated Tregs, arguing against active CD131 signaling on Tregs. Likewise, EPO-R, which can also heterodimerize with CD131, was hardly expressed on Tregs. Since *IL3RA* showed the highest mRNA levels within the studied markers in activated Tregs and high protein expression has been reported in murine Tregs (18), we analyzed the IL-3RA protein expression pattern under different environmental stimuli in detail. Previous studies described IL-3RA upregulation on human T cells after *in vitro* culture in the presence of IL-2 or IL-4, while IL-10 restrained its expression (34, 35). We hypothesized that, alike Tconvs, Tregs may adapt IL-3RA expression in response to their cytokine environment. However, despite our efforts, we did not identify the presence of a distinct CD131^+^IL-3RA^+^ Treg subset after culturing with pro- or anti-inflammatory cytokines (IL-4, IL-1β, IL-6 or TGFβ and IL-10 respectively). Further studies are warranted in order to identify whether CD131^+^IL-3RA^+^ Tregs are present under certain physiological conditions. It is also possible that CD131 may have a role in Treg function beyond its association with the alpha-chains of IL-3, GM-CSF and IL-5 receptors. However, *CSF2RB*-KO in human Tregs showed unaltered phenotype and maintained *in vitro*-suppressive capacity to a similar extent than controls.

Despite the challenge to establish a functional significance for *CSF2RB* in human Tregs, a major limitation of our *in vitro* data is that it does not accurately represent *in vivo* Treg conditions. Tregs are highly adaptable according to specific environmental conditions and energy demands (43) and, therefore, *in vitro* data may so provide only limited information about regulation and function of *CSF2RB* and associated molecules. Thus, further studies are necessary to fully characterize any potential role *CSF2RB* might exert in Treg stability and function and its role in autoimmune disorders. This would be of particular interest since its potential ligands, the β common chain cytokines, play a key role in several inflammatory processes and autoimmunity (44).

In summary, our data demonstrate that human Tregs of healthy donors constitutively transcribe *CSF2RB*, but protein expression was much higher after TCR stimulation. Despite detectable CD131 protein expression, STAT5 phosphorylation, measured as a readout of CD131 intracellular signaling, was not induced in Tregs upon treatment with β common chain cytokines. Moreover, Treg phenotype and *in vitro* suppressive capacity were unaltered in *CSF2RB*-KO Tregs. Collectively, our data did not identify an active role for *CSF2RB* in human Tregs. Of note, we did not observe co-expression of CD131 and any other of the alpha-chain receptors (IL3-RA, IL-5RA, GMR-α or EPO-R) on Tregs, suggesting that CD131 function may be regulated by tight expression of the alpha subunits. Further, guided by previous genetic and transcriptomic studies, we demonstrate an association between *CSF2RB* expression in Tregs and autoimmunity. *CSF2RB* has susceptibility variants for MS and *CSF2RB* is significantly overexpressed in Tregs of MS and SLE patients compared to healthy controls. This may qualify *CSF2RB* expression of Tregs as a novel biomarker for disease. However, future studies are needed to verify these results in larger patient cohorts.

## Data availability statement

The data that support the findings of this study are available from the corresponding author upon reasonable request. Publicly available datasets were analyzed in this study, the names of the repositories/accession numbers are listed in the article/**Supplementary Materials**.

## Ethics statement

The studies involving human participants were reviewed and approved by institutional ethics committee of Hasselt University (Comité voor Medische Ethiek UHasselt, CME2019/042 and CME2016/629). The patients/participants provided their written informed consent to participate in this study.

## Author contributions

BFC-R, RAH, AD, and IH designed and performed experiments, analyzed and interpreted the data and wrote the manuscript. MK led and conceived the project, supervised experiments, interpreted data and wrote the manuscript. All authors contributed to the article and approved the submitted version.

## Funding

MK was supported by the European Research Council (ERC) under the European Union’s Horizon 2020 research and innovation program (640116), by a SALK-grant from the government of Flanders, by an Odysseus-grant (G0G1216FWO) and senior research project (G080121N) of the Research Foundation Flanders, Belgium (FWO) and by a BOF grant (ADMIRE, 21GP17BOF) from Hasselt University.

## Acknowledgments

We thank Dries Swinnen for technical assistance.

## Conflict of interest

The authors declare that the research was conducted in the absence of any commercial or financial relationships that could be construed as a potential conflict of interest.

## Publisher’s note

All claims expressed in this article are solely those of the authors and do not necessarily represent those of their affiliated organizations, or those of the publisher, the editors and the reviewers. Any product that may be evaluated in this article, or claim that may be made by its manufacturer, is not guaranteed or endorsed by the publisher.

## Supplementary material

Supplementary Figure 1 Phenotype of FACS-sorted CD4^+^ Tconv and Tregs. Representative reanalysis of CD4, CD25, CD127 and FOXP3 phenotype of peripheral blood isolated CD4^+^ Tconv and Tregs.

Supplementary Figure 2
*CSF2RB* epigenetic and transcriptomic signature. **(A)** Epigenetic signature at the *CSF2RB* locus was studied using WashU epigenome browser (v52.1.0) (27). Data from human CD4^+^CD25^-^ Th and CD4^+^CD25^+^CD127^-^ Treg primary cells obtained from blood of healthy donors were downloaded from the Roadmap Epigenomics Project (26, 45). *CSF2RB* gene track from gencodeV29 is shown in purple. The chromatin state (chromHMM) and histone marks (H3K4me1, H3K4me3, H3K36me3, H3K27me3 and H3K9me3) at the *CSF2RB* locus are shown for Th (black line) and Tregs (red line). **(B)** *CSF2RB* mRNA expression analyzed in PBMC subsets. Data were obtained from (http://www.proteinatlas.org).

Supplementary Figure 3 Re-analysis of *CSF2RB* mRNA expression on CD4^+^ Tconv and Treg populations. mRNA expression was studied in published Treg signature data. **(A)** data-set from GSE90600 (11) or **(B, C)** from GSE76598 (10). **(A)** RNAseq was performed in different CD4^+^ T cell subsets sorted from blood of 3 healthy donors based on CD4, CD25 and CD45RA expression. CD4^+^ T cells were isolated as: CD45RA^+^CD25^-^ naive T cells (nTconv), CD45RA^-^CD25^-^ memory T cells (mTconv), CD45RA^-^CD25^int^, CD45RA^+^CD25^+^ naive Tregs (nTreg) and CD45RA^-^ CD25^high^ effector Tregs (eTreg). **(B, C)** Cells were sorted from blood of 7 healthy donors as: CD4^+^CD45RA^+^CD25^-^ naive T cells (nTconv), CD4^+^CD45RA^-^CD25^-^ memory T cells (mTconv), CD4^+^CD45RA^+^ CD25^+^ naive Tregs (nTreg) and CD4^+^CD45RA^-^CD25^+^ memory Tregs (mTreg). RNA expression was studied in **(B)** *ex vivo* cell subsets or **(C)** after 40 hours of stimulation with anti-CD3 and anti-CD28 antibodies. Significance was calculated by **(A, B)** one-way ANOVA with Sidak’s multiple comparisons test or **(C)** Kruskal-Wallis test with Dunn’s multiple comparisons test.

Supplementary Figure 4 Re-analysis of expression of CD131-associated cytokine receptors on CD4^+^ Tconv and Treg populations. mRNA expression was studied in published Treg signature data **(A)** GSE90600 (11) or **(B, C)** GSE76598 (10). **(A)** RNAseq was performed in cells sorted from blood of 3 healthy donors based on CD4, CD25 and CD45RA expression. CD4^+^ T cells were isolated as: CD45RA^+^CD25^-^ naive T cells (nTconv), CD45RA^-^CD25^-^ memory T cells (mTconv), CD45RA^-^CD25^int^, CD45RA^+^CD25^+^ naive Tregs (nTreg) and CD45RA^-^ CD25^high^ effector Tregs (eTreg). **(B, C)** Cells were sorted from blood of 7 healthy donors as: CD4^+^CD45RA^+^CD25^-^ naive T cells (nTconv), CD4^+^CD45RA^-^CD25^-^ memory T cells (mTconv), CD4^+^CD45RA^+^ CD25^+^ naive Tregs (nTreg) and CD4^+^CD45RA^-^CD25^+^ memory Tregs (mTreg). RNA expression was studied in **(B)** *ex vivo* cell subsets or **(C)** after 40 hours of stimulation with anti-CD3 and anti-CD28 antibodies. Significance was calculated by one-way ANOVA with Sidak’s multiple comparisons test for normally-distributed data or by a Kruskal-Wallis test with Dunn’s multiple comparisons test for data non-normally distributed.
