# Supplementary material for: Dissecting the role of CSF2RB expression in human regulatory T cells
Source: Front Immunol. 2022 Dec 2;13:1005965. doi: 10.3389/fimmu.2022.1005965 (PMC9755334; doi:10.3389/fimmu.2022.1005965)
Supplement: Supplementary file 1 [file Table_1.pdf]

Supplementary tables

Table S1: gRNA sequence for CSF2RB

**gRNA sequence + (PAM)**	**Exon targeted**
CATCTCCTGGGCACGCAGCG (GGG)	*4*

Table S2: Overview linkage of CSF2RB to disease/phenotype (GWAS Catalog)

**PUBMED ID**	**First Author**	**Journal**	**Study**	**Disease/Trait**
22138694	Lin Z	Nat Genet	A genome-wide association study in Han Chinese identifies new susceptibility loci for ankylosing spondylitis.	Ankylosing spondylitis
28199695	Jones AV	Hum Mol Genet	GWAS of self-reported mosquito bite size, itch intensity and attractiveness to mosquitoes implicates immune-related predisposition loci.	Itch intensity from mosquito bite adjusted by bite size
28199695	Jones AV	Hum Mol Genet	GWAS of self-reported mosquito bite size, itch intensity and attractiveness to mosquitoes implicates immune-related predisposition loci.	Mosquito bite size
28199695	Jones AV	Hum Mol Genet	GWAS of self-reported mosquito bite size, itch intensity and attractiveness to mosquitoes implicates immune-related predisposition loci.	Perceived unattractiveness to mosquitoes
28199695	Jones AV	Hum Mol Genet	GWAS of self-reported mosquito bite size, itch intensity and attractiveness to mosquitoes implicates immune-related predisposition loci.	Itch intensity from mosquito bite
26920376	Wang Y	Mult Scler	Genetic overlap between multiple sclerosis and several cardiovascular disease risk factors.	Multiple sclerosis and LDL levels (pleiotropy)
31604244	IMSGC	Science	Multiple sclerosis genomic map implicates peripheral immune cells and microglia in susceptibility.	Multiple sclerosis
32888494	Vuckovic D	Cell	The Polygenic and Monogenic Basis of Blood Traits and Diseases.	Eosinophil percentage of white cells
31361310	Johansson A	Hum Mol Genet	Genome-wide association analysis of 350.000 Caucasians from the UK Biobank identifies novel loci for asthma, hay fever and eczema.	Hay fever and/or eczema
32888494	Vuckovic D	Cell	The Polygenic and Monogenic Basis of Blood Traits and Diseases.	Eosinophil counts
29875488	Sun BB	Nature	Genomic atlas of the human plasma proteome.	Blood protein levels
30072576	Emilsson V	Science	Co-regulatory networks of human serum proteins link genetics to disease.	Blood protein levels
24076602	Beecham AH	Nat Genet	Analysis of immune-related loci identifies 48 new susceptibility variants for multiple sclerosis.	Multiple sclerosis
27569725	Yang SK	Gastroenterology	Identification of Loci at 1q21 and 16q23 That Affect Susceptibility to Inflammatory Bowel Disease in Koreans.	Inflammatory bowel disease
32888494	Vuckovic D	Cell	The Polygenic and Monogenic Basis of Blood Traits and Diseases.	Eosinophil percentage of white cells
32888493	Chen MH	Cell	Trans-ethnic and Ancestry-Specific Blood-Cell Genetics in 746,667 Individuals from 5 Global Populations.	Basophil count
29679657	Ferreira MA	J Allergy Clin Immunol	Eleven loci with new reproducible genetic associations with allergic disease risk.	Allergic disease (asthma, hay fever or eczema)
