# Supplementary figures and images for: Dissecting the role of CSF2RB expression in human regulatory T cells
Source: Front Immunol. 2022 Dec 2;13:1005965. doi: 10.3389/fimmu.2022.1005965 (PMC9755334; doi:10.3389/fimmu.2022.1005965)

Fig. S1

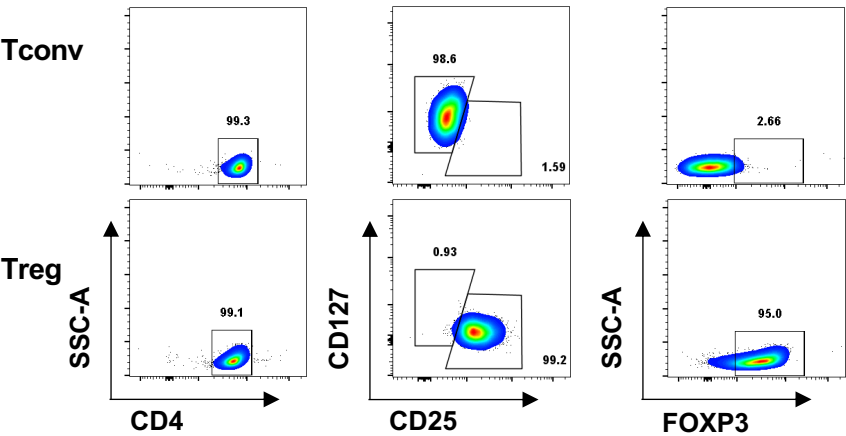

Fig. S2

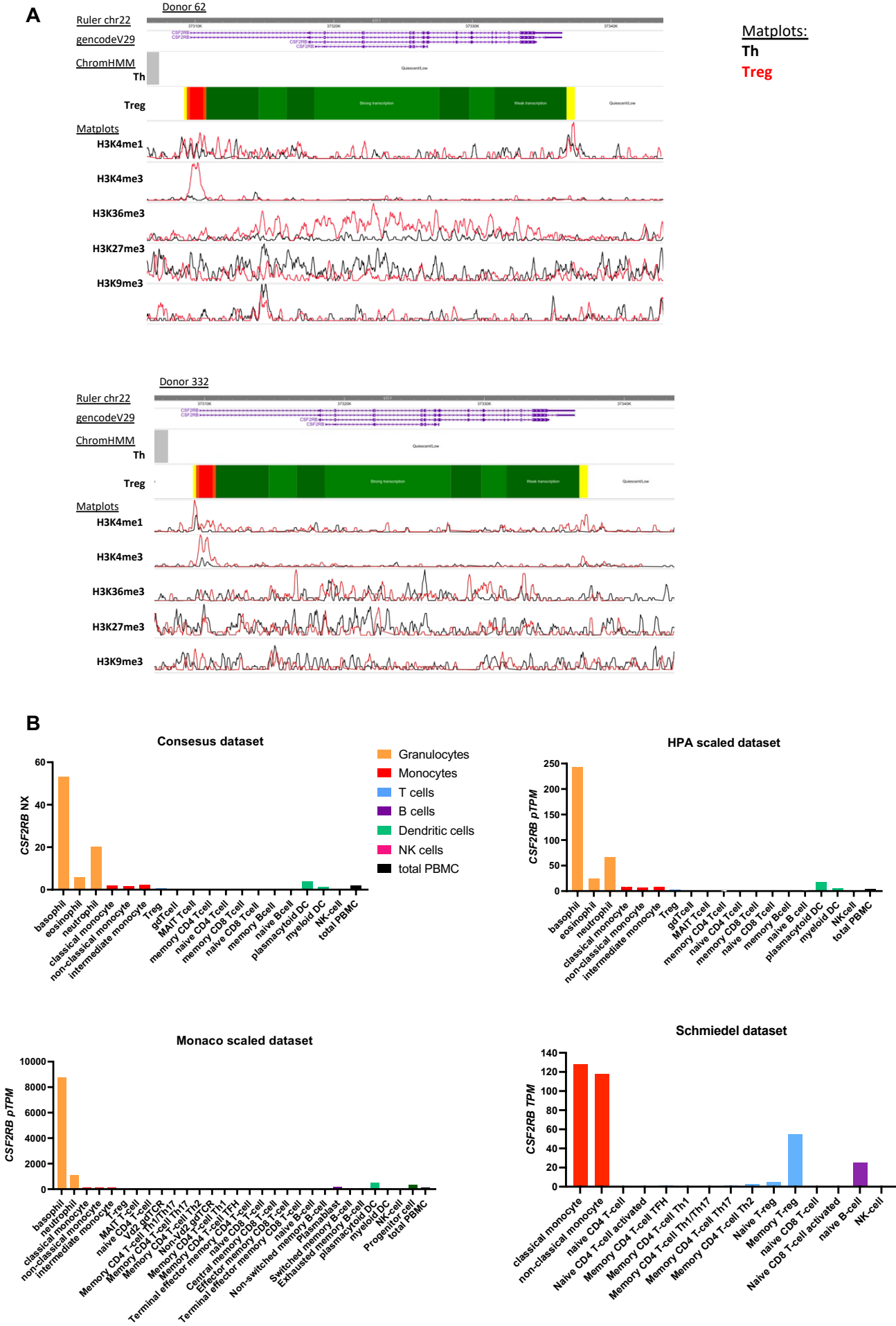

Fig. S3

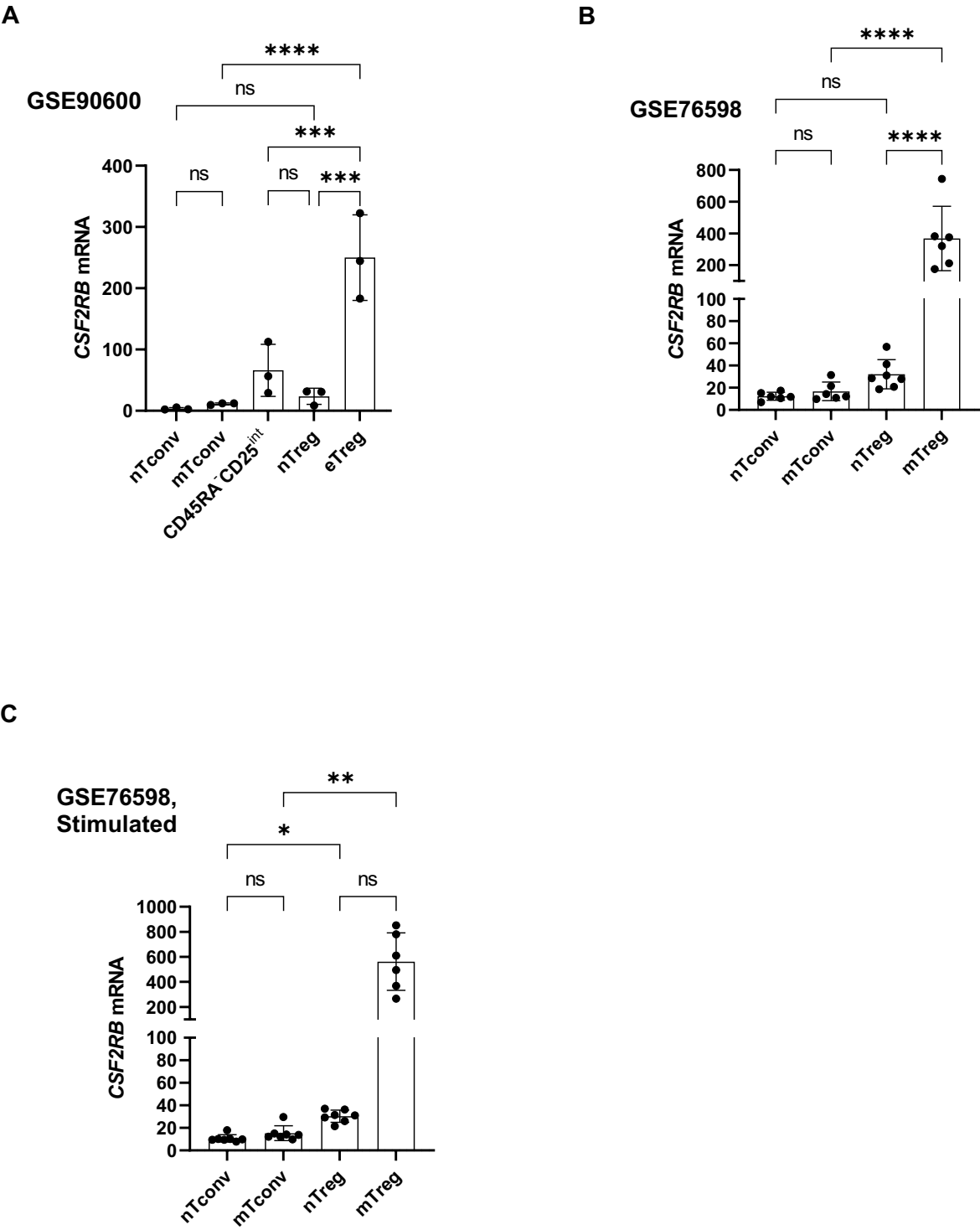

**Fig. S4**

**A**

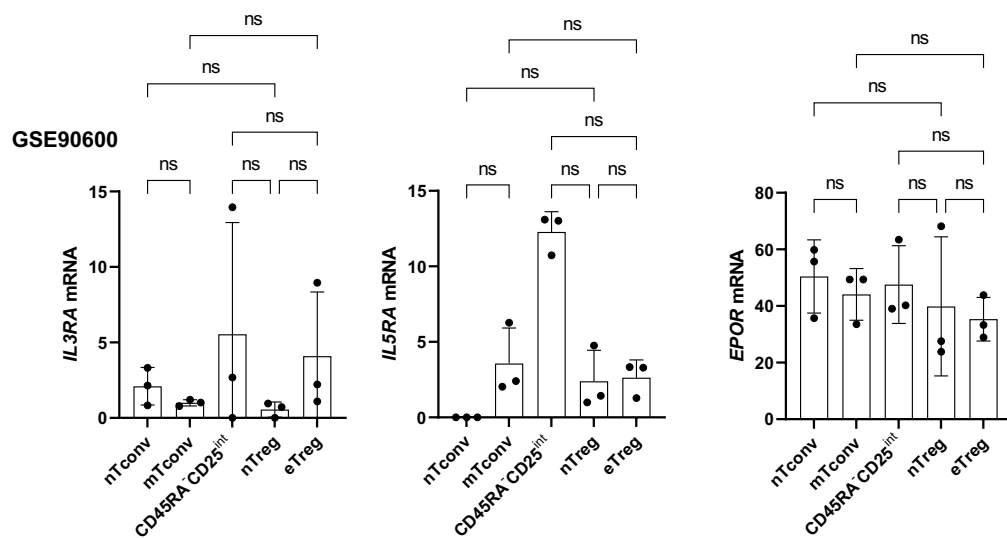

**B**

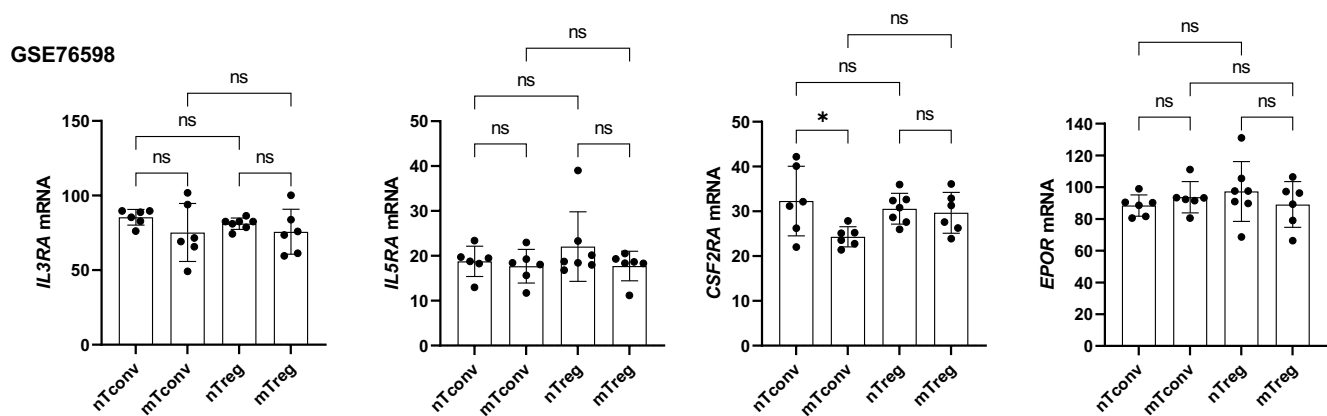

**C**

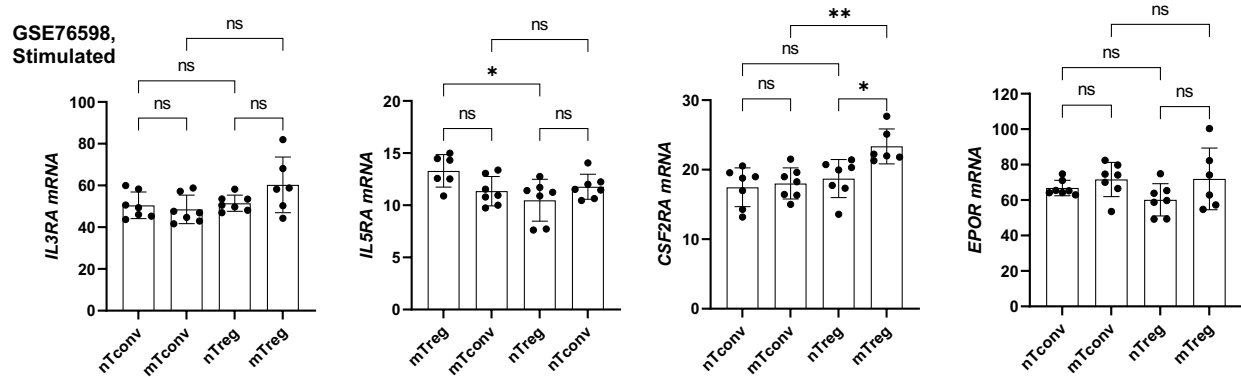

Supplement: Supplementary Figure 1 — Phenotype of FACS-sorted CD4+ Tconv and Tregs. Representative reanalysis of CD4, CD25, CD127 and FOXP3 phenotype of peripheral blood isolated CD4+ Tconv and Tregs. [file DataSheet_1.pdf]
